# MiR-138-5p Suppresses Cell Growth and Migration in Melanoma by Targeting Telomerase Reverse Transcriptase

**DOI:** 10.3390/genes12121931

**Published:** 2021-11-30

**Authors:** Estefanía Tarazón, Blanca de Unamuno Bustos, Rosa Murria Estal, Gema Pérez Simó, Antonio Sahuquillo Torralba, Javier Simarro, Sarai Palanca Suela, Rafael Botella Estrada

**Affiliations:** 1 Dermatology and Tisular Regeneration Group, Health Research Institute La Fe, 46026 Valencia, Spain; tarazon_est@gva.es (E.T.); blancaunamuno@yahoo.es (B.d.U.B.); romues@alumni.uv.es (R.M.E.); saucodos@gmail.com (A.S.T.); rbotellaes@gmail.com (R.B.E.); 2 Department of Dermatology, University Hospital La Fe, 46026 Valencia, Spain; 3 Clinical and Translational Cancer Research Group, Health Research Institute La Fe, 46026 Valencia, Spain; gema_perez_s@hotmail.com (G.P.S.); javier_simarro@iislafe.es (J.S.); 4 Laboratory of Molecular Biology, Service of Clinical Analysis, University Hospital La Fe, 46026 Valencia, Spain; 5 Department of Medicine, School of Medicine, Universitat de València, 46010 Valencia, Spain

**Keywords:** melanoma, hTERT, miR-138-5p

## Abstract

Recent evidence suggests the existence of a miRNA regulatory network involving human telomerase reverse transcriptase gene (*hTERT*), with miR-138-5p playing a central role in many types of cancers. However, little is known about the regulation of *hTERT* expression by microRNA (miRNAs) in melanocytic tumors. Here, we investigated the effects of miR-138-5p in *hTERT* regulation in melanoma cells lines. In vitro studies demonstrated higher miR-138-5p and lower *hTERT* messenger RNA (mRNA) expression in human epidermal melanocytes, compared with melanoma cell lines (A2058, A375, SK-MEL-28) by quantitative polymerase chain reaction (qPCR) observing a negative correlation between them. A2058 melanoma cells were selected to be transfected with miR-138-5p mimic or inhibitor. Using luciferase assay, *hTERT* was identified as a direct target of this miRNA. Overexpression of miR-138-5p detected by Western blot revealed a decrease in hTERT protein expression (*p* = 0.012), and qPCR showed a reduction in telomerase activity (*p* < 0.001). Moreover, suppressions in cell growth (*p* = 0.035) and migration abilities (*p* = 0.015) were observed in A2058-transfected cells using thiazolyl blue tetrazolium bromide and flow cytometry, respectively. This study identifies miR-138-5p as a crucial tumor suppressor miRNA involved in telomerase regulation. Targeting it as a combination therapy with immunotherapy or targeted therapies could be used in advanced melanoma treatment; however, more preclinical studies are necessary.

## 1. Introduction

Melanoma is considered the most dangerous form of skin cancer. Although it represents less than 5% of all cutaneous neoplasms, it is responsible for almost 60% of deaths from skin tumors [1], and its incidence has rapidly increased over the past three decades [2]. When melanoma primary arises on mucosal surfaces, the outcome of the condition and overall survival may be even worse [3].

The human telomerase reverse transcriptase gene (*hTERT*) has been implicated in melanoma pathogenesis [4,5]. This gene encodes the catalytic subunit of telomerase that is essential for telomere maintenance and cell senescence regulation. *TERT* promoter mutations (pTERTm) have been described in sporadic melanoma at a high frequency (20–70%) and have been associated with aggressive clinicopathological features and poor prognosis [6,7,8,9]. Functional studies have demonstrated that pTERTm enhance *hTERT* mRNA expression by creating de novo binding motifs for the ETS transcription factors [4,10,11]. Nevertheless, previous results of our group, as well as those of others, have not identified differences at a protein level between pTERTm carriers and noncarrier tumors [9,12,13,14,15].

In this regard, other mechanisms such as *hTERT* gene amplification [16], promoter activators [17], or DNA methylation [18,19,20] have been suggested to be responsible for telomerase activation in non-pTERTm carriers. Additionally, recent studies suggest the existence of a microRNA (miRNA) regulatory network involving *hTERT* [20,21]. Hrdličková et al. showed that multiple miRNAs target the *hTERT* 3′UTR using luciferase reporter screens such as let-7g, miR-133a, miR-138-5p, miR-342-5p, miR-491-5p, and miR-541-3p [21]. MiRNAs are a type of noncoding RNA that regulate gene expression at the posttranscriptional level by degrading their target mRNAs or inhibiting protein translation by directly binding to the 3′-untranslational region of mRNAs [22,23]. Increasing evidence shows that miRNAs are involved in the control of a wide variety of biological processes, including cell proliferation, differentiation, survival, apoptosis, and cell cycle progression [24,25,26,27]. Regarding melanocytic lesions, previous studies have revealed different miRNAs expression profiles between melanoma and benign nevi [28], as well as specific expression patterns associated with melanoma progression and invasion [29]. However, to date, few studies have analyzed the regulation of *hTERT* expression by miRNAs in melanocytic tumors identifying miR-497-5p, miR-195-5p, and miR-455-3p as tumor suppressors by targeting *hTERT* in melanoma A375 cells [30]. Additionally, miR-19b has also been implicated in *TERT* mRNA expression regulation through targeting PITX1 in melanoma cells [31]. Additionally, the regulation of *hTERT* by miR-138-5p has been described in human cervical cancer [32], colorectal cancer [33,34], and thyroid carcinoma cell lines [35].

Therefore, the aim of this study was to explore the role of miR-138-5p as a tumor suppressor and their implication in the regulation of *hTERT* in human melanoma through functional studies in melanoma cells lines.

## 2. Materials and Methods

### 2.1. Cell Lines

Human melanoma A2058, A375, and SK-MEL-28 cells were purchased from the ATCC and cultured in DMEM medium (Gibco, Thermo Fisher Scientific, Carlsbad, CA, USA), supplemented with 10% fetal bovine serum (FBS), 100 U/mL penicillin, and 100 µg/mL streptomycin, at 37 °C in a 5% CO_2_ incubator. Human epidermal melanocytes were kindly provided by Dr. Julián Carretero (Universitat de València, Valencia, Spain), and cultured in 254CF medium supplemented with calcium chloride at a final concentration of 0.2 mM, with a human melanocyte growth supplement (Gibco, Thermo Fisher Scientific, Carlsbad, CA, USA), 100 U/mL penicillin and 100 µg/mL streptomycin, at 37 °C in a 5% CO_2_ incubator. The medium was changed daily.

### 2.2. Detection of pTERTm

The mutational status of the *TERT* promoter region (LRG_343t1) was determined by polymerase chain reaction (PCR) and Sanger sequencing. PCR was performed using previously described primers of the *TERT* promoter: hTERT_2F CTCCCAGTGGATTCGCGGGC and hTERT_2R CCCACGTGCGCAGCAGGAC (260bp product) [8]. The PCR was performed in an automatic DNA thermal cycler using the following cycling conditions: initial heating at 95 °C for 5 min, followed by 40 cycles at 95 °C for 45 s, 60 °C for 45 s, 72 °C for 36 s, and, finally, 72 °C for 10 min. Amplified products were purified with Exosap (GE Healthcare, Buckinghamshire, UK) and direct sequencing was performed on a capillary sequencer (AbiPrism 3130xl Genetic Analyzer).

### 2.3. Cell Transfection and Molecule Extractions

In total, 5 × 10^4^ human melanoma cells per well were seeded in 24-well plates and were transfected with miR-138-5p mimic (5′-AGCUGGUGUUGUGAAUCAGGCCG-3′) or negative control at 50 nM (ID: 1027280 and 219600 from Qiagen, Düsseldorf, Germany), after 24 h being cultured (≥80% confluence) using TransIT-siQUEST (Mirus Bio, Madison, Wisconsin, United States) in serum-free medium (Opti-MEMI Reduced-Serum Medium, Thermo Fisher Scientific, Carlsbad, California, United States) according to the manufacturer’s instructions. At 48 h posttransfection, the cells were harvested, and the expression of the target molecules was analyzed.

Total cells’ RNA was extracted using the TRIZOL method and was quantified using a Qubit Fluorometer (Thermo Fisher Scientific, Carlsbad, CA, USA). For protein isolation, cells were treated with total protein extraction buffer (50 mM Tris-HCl, 50 mM NaCl, 0.5 mM EGTA, 1 mM EDTA, 0,1% SDS, 1% Triton X-100, pH 7.5) supplemented with protease inhibitors (25 µg/mL aprotinin and 10 µg/mL leupeptin). Samples were sonicated and total protein was quantified by the Bradford method (Coomassie (Bradford) Protein Assay Kit, Thermo Fisher Scientific, Carlsbad, CA, USA).

### 2.4. Luciferase Assay

In total, 7.5 × 10^3^ cells per well were seeded into 96-well plates and cotransfected (≥80% confluence) with the designated luciferase reporter plasmid (*TERT*, LightSwitch™ 3′ UTR Reporter GoClone^®^, Active Motif, Waterloo, Belgium) and with miR-138-5p mimic (QIAGEN, Düsseldorf, Germany) or negative control with Lipofectamine^®^ 3000 Transfection Kit (Invitrogen, Waltham, MA, USA). GAPDH and random C1 luciferase reporter plasmids were included as internal controls for transfection efficiency (Supplemental Tables S1–S3). The interaction between miR-138-5p and *TERT* was measured by comparing the results of the cotransfection of the *TERT* 3′ UTR–luciferase reporter and miR-138-5p mimic with those of the 3′ UTR–luciferase reporter plasmid with negative control. The luciferase assay was performed using the LightSwitch™ Luciferase Assay Kit (Active Motif, Waterloo, Belgium).

### 2.5. Cell Proliferation Assays

Cell proliferation assay was determined by using thiazolyl blue tetrazolium bromide (MTT) from Sigma-Aldrich (Saint Louis, MO, USA). A total of 7.5 × 10^3^ cells per well with miRNA mimic or negative control were seeded into 96-well plates and cultured for 48 h. Then, 10 µL MTT solutions (5 mg/mL) were added to each well and incubated at 37 °C in a 5% CO2 incubator for 3 h. After removing the culture medium, 100 µL DMSO (Sigma-Aldrich, Saint Louis, MO, USA) was added to each well mixing well. The optical density values at 560 nm were measured with a microplate reader (Promega, Madison, WI, USA). All the experiments were performed in triplicate.

### 2.6. Cell Cycle Assays

In total, 5 × 10^4^ cells per well with miRNA mimic or negative control were seeded into 24-well plates and cultured for 48 h. Cells were harvested and fixed with cold ethanol for 2 h. Then, they were stained with propidium iodide protected from light at 4 °C for 30 min. Cell cycle status was measured by flow cytometry (Beckman Coulter). All the experiments were performed in triplicate.

### 2.7. Telomerase Activity

Telomerase activity was determined by using Telomerase Activity Quantification qPCR Assay Kit (ScienCell Research Laboratories, Carlsbad, CA, USA) according to the manufacturer’s instructions. A total of 5 × 10^4^ cells per well with miRNA mimic or negative control were seeded into 24-well plates and cultured for 48 h. After lysing cells, the telomerase reactions were prepared for each sample and incubated at 37 °C for 3 h. The reaction was stopped by heating the samples to 85 °C for 10 min and the quantitative PCR (qPCR) was performed on the LightCycler^®^ 480 II Instrument (Roche, Basel, Switzerland). All the experiments were performed in triplicate.

### 2.8. Cell Migration Assay

A wound healing assay was performed to test the migration ability of A2058 cells. For this assay, 5 × 10^4^ cells per well with miRNA mimic or negative control were seeded into 24-well plates and cultured for 48 h. Scratches were performed with 100 µL pipette tips and were monitored with an inverted microscope (Leica DMi8 platform) for live cells for 29 h. The ImageJ program was used to measure the area of the wound after 12 and 29 h of follow-up. The migration ability was calculated as the % of the healing area with respect to time 0.

### 2.9. Western Blot Assay

Proteins were separated using Mini-PROTEAN TGX gels with 10% polyacrylamide (Bio-Rad). After electrophoresis, the proteins were transferred from the gel to a nitrocellulose membrane by wet transfer using the Mini Trans-Blot^®^ module (Bio-Rad, Hercules, CA, USA). The membranes were blocked by 1×TBST solution containing 45% nonfat milk for 1 h at room temperature and incubated with primary anti-Telomerase reverse transcriptase antibody-C-terminal (1:1000) and anti-β Actin antibody (1:2000) from Abcam (Cambridge, UK) overnight at 4 °C. The bands were visualized using a secondary antibody conjugated to horseradish peroxidase (HRP) and Pierce™ ECL Western blotting substrate (Thermo Fisher Scientific, Carlsbad, CA, USA), according to the manufacturer’s instructions. ImageJ software was used to quantify the integrated density of the band. All the experiments were performed in triplicate.

### 2.10. Real Time qPCR (RT-qPCR)

MiR-138-5p and *hTERT* mRNA expression were performed employing the miScript II Reverse Transcription Kit, miScript Primer Assay, and miScript SYBR Green PCR Kit (for miR-138-5p, QIAGEN, Düsseldorf, Germany) or TaqMan^®^ Gene Expression Assays (for *hTERT*, Thermo Fisher Scientific, Carlsbad, CA, USA) with the 7500 real-time PCR system (Applied Biosystems, Waltham, MA, USA). All experiments were carried out in duplicate, normalization was performed regarding the small nuclear SNORD95 and GAPDH, and relative expression was calculated using the 2^−ΔΔCt^ method [36].

### 2.11. Statistics

Data are expressed as the mean ± standard deviation. The Kolmogorov–Smirnov test was applied for analyzing the cells’ data distribution. Significant mean differences between groups with a normal distribution were analyzed by using Student’s *t*-test, whereas the nonparametric Mann–Whitney U test was performed for comparisons between data that were non-normally distributed. *p* < 0.05 was considered statistically significant. All statistical analyses were performed using the SPSS software (version 20.0) for Windows (IBM SPSS Inc., New York, NY, USA).

## 3. Results

### 3.1. Mutational Characterization of hTERT Promoter and hTERT and miR-138-5p Expression in Melanoma Cell Lines and Human Epidermal Melanocytes

Mutational characterization showed that the A2058 and A375 cell lines had the most frequent pTERTm: c.−124C > T and c.−146C > T, respectively. However, the SK-MEL-28 cell line and human epidermal melanocytes were wild types for these mutations.

**Figure 1:**
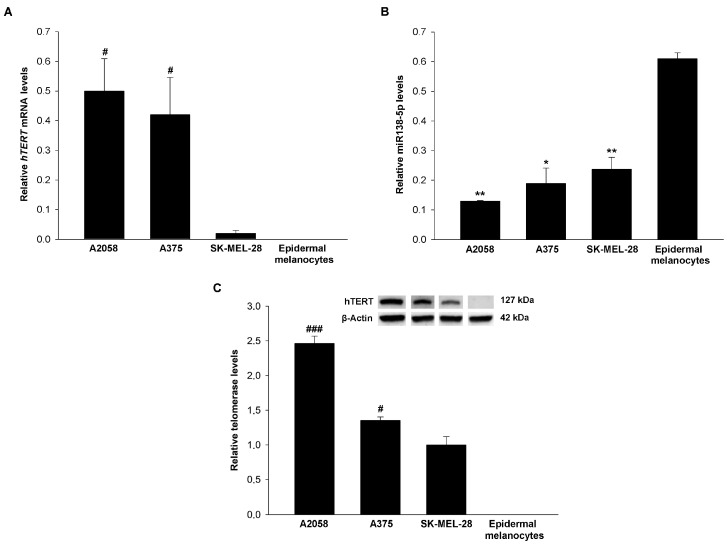
Human telomerase reverse transcriptase (hTERT) mRNA, miR-138-5p, and telomerase protein levels in melanoma cell lines and human epidermal melanocytes: (**A**) relative hTERT mRNA, (**B**) miR-138-5p, and (**C**) telomerase levels in A2058, A375, and SK-MEL-28 melanoma cells, and human epidermal melanocytes. # *p* < 0.05 and ### *p* < 0.001 vs. SK-MEL-28, and * *p* < 0.05 and ** *p* < 0.01 vs. human epidermal melanocytes. Relative messenger RNA (mRNA) and microRNA (miRNA) expressions were performed by Real Time quantitative polymerase chain reaction (RT-qPCR) in a 7500 real-time PCR system using small nuclear SNORD95 and GAPDH as normalizers. Relative expression was calculated using 2^−ΔΔCt^ method. Telomerase protein levels were determined by Western blot. β-Actin was used as protein normalizer.

As shown in Figure 1A, the expression of mRNA hTERT was greater in the melanoma cell lines with −124C > T and −146C > T mutations, compared with the SK-MEL-28 cell line. Expression was not detectable in human epidermal melanocytes with the methodology used. The expression of miR-138-5p showed a negative correlation (r = −0.733) with mRNA hTERT expression so that human epidermal melanocytes showed the highest expression, significantly higher than A2058, A375, and SK-MEL-28 (Figure 1B). These results were confirmed by measuring protein levels of hTERT by Western blot (Figure 1C).

Given these levels of hTERT, miR-138-5p, and telomerase, we selected cell line A2058 (−124C > T) for further studies.

### 3.2. Effect of miR-138-5p Overexpression on Melanoma Cells

**Figure 2:**
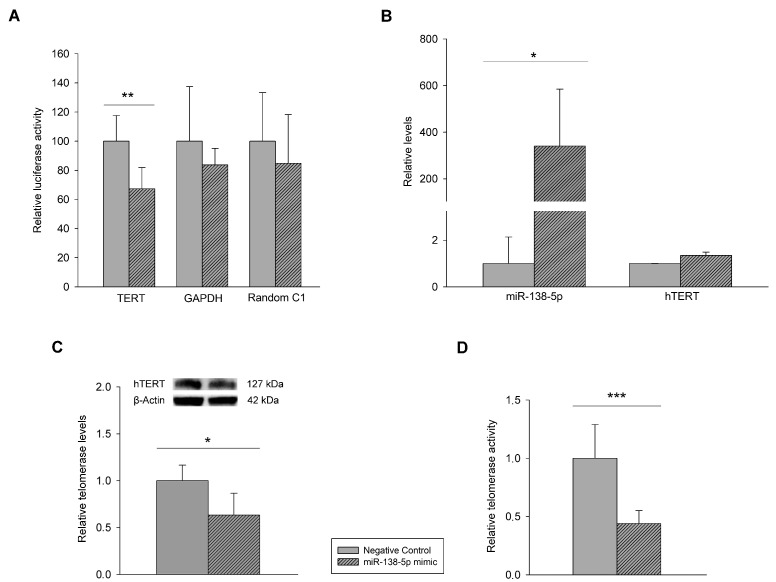
Induction of miR-138-5p in A2058 melanoma cells: (**A**) luciferase reporter assay performed using the LightSwitch™ Luciferase Assay System. The data from housekeeping (GAPDH) and random (C1) constructs were used to control for non-UTR specific treatment effects; (**B**) miR-138-5p transfection efficiency and human telomerase reverse transcriptase (hTERT) levels determined by Real Time quantitative polymerase chain reaction (RT-qPCR) as in Figure 1; (**C**) effects on TERT protein expression using Western blot, the data are relative to negative control group whose values were set to 1; (**D**) telomerase activity determined by using Telomerase Activity Quantification qPCR Assay Kit by qPCR. The data are relative telomerase activity to negative control group based on 2^−ΔCq^ of Cq obtained with LightCycler^®^ 480 II Instrument. Bars with striped patterns are miR-138-5p mimic group, and bars with and smooth patterns are negative control group. * *p* < 0.05, ** *p* < 0.01, *** *p* < 0.001.

Measurement of luciferase activity showed that the signal from human 3′UTR reporter for hTERT gene was significantly knocked down in the presence of the miR-138-5p mimic, while signals for housekeeping and random sequence UTRs were not (Figure 2A), demonstrating that hTERT was the target of miR-138-5p. The upregulation of miR-138-5p (Figure 2B) did not modify hTERT mRNA expression in our cells, but miR-138-5p was able to block hTERT translation, significantly decreasing hTERT protein expression (Figure 2C). Moreover, miR-138-5p upregulation resulted in a reduction over twofold the telomerase activity (FC = −2.21, *p* = 0.001) of A2058 cells.

**Figure 3:**
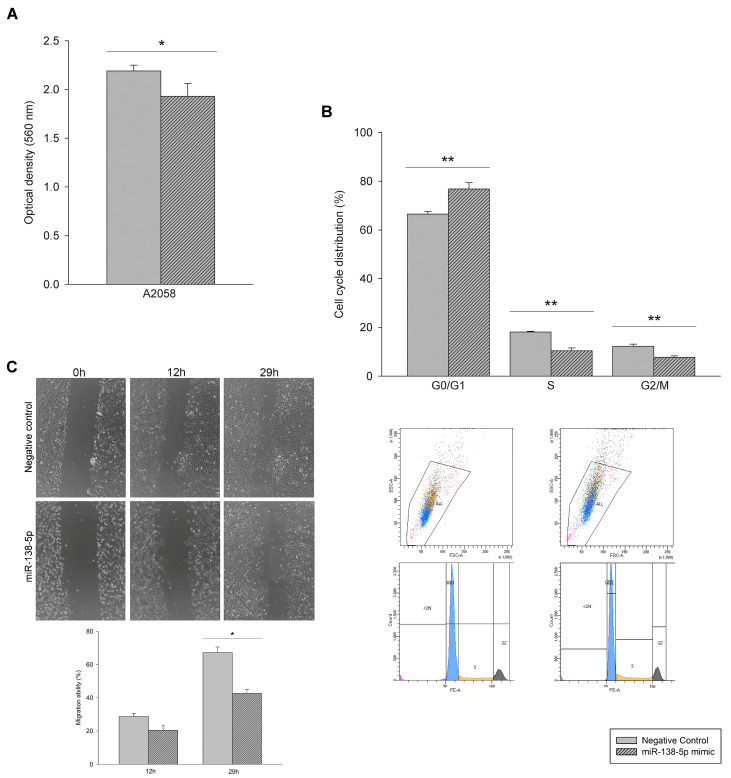
Inhibition of the proliferation and migration of A2058 melanoma cells by miR-138-5p overexpression. Cell proliferation was determined by spectrophotometric quantification at 560 nm using thiazolyl blue tetrazolium bromide (MTT) (**A**), cell cycle distribution was measured by flow cytometry using propidium iodide to stain (**B**), and migration ability by wound healing assay, monitoring cells with Leica DMi8 platform for 29 h. The migration ability was calculated as the % of healing area with respect to time 0 (**C**). Bars with striped patterns are miR-138-5p mimic group, and bars with and smooth patterns are negative control group. * *p* < 0.05, ** *p* < 0.01.

Likewise, the miR-138-5p mimic transfection suppressed cells growth, observing significantly lower proliferation (Figure 3A) and cell arrest in G0/G1 phase. As shown in Figure 3B, cells of the G0/G1 phase were significantly higher in the miR-138-5p-transfected group than in the control group, while the S and G2/M phase cells were decreased. Further, miR-138-5p overexpression suppressed A2058 migration abilities (Figure 3C).

## 4. Discussion

Different miRNAs have been described as important regulators of *hTERT* in multiple types of malignant tumors. *HTERT*-targeting miRNAs mediate the posttranscriptional gene silencing inducing translation repression or mRNA degradation, thus inhibiting the aberrant self-renewal capacity of cancer cells [21,37]. To date, however, little is known about the role of miRNAs in melanoma *hTERT* regulation. This study identified miR-138-5p as a crucial tumor suppressor miRNA involved in telomerase regulation.

Downregulation of miR-138-5p has been frequently observed in various cancers and highly metastatic cells [38]. Liu et al. identified miR-138-5p downregulation in human head and neck squamous cell carcinoma cell lines and demonstrated that overexpression was associated with an inhibition of proliferation through cell cycle arrest and apoptosis and with suppression of tumoral cells invasion [39]. Chen et al. also found that miR-138-5p was downregulated in ovarian cancer and that the overexpression of miR-138-5p inhibited ovarian cancer cell proliferation, migration, and invasion [40]. Moreover, miR-138-5p has been described as downregulated in colorectal cancer, decreasing proliferation and promoting apoptosis by targeting 3′UTR of *hTERT* [27]. In addition, Mitomo et al. identified miR138-5p downregulation in human anaplastic thyroid carcinoma cell lines and described *hTERT* as a direct target of miR-138, with a reduction in hTERT protein expression mediated by the enforced overexpression of miR-138 [35].

Regarding melanoma, only two studies have analyzed miR-138-5p expression. Chen et al. identified miR-138-5p downregulation in melanoma WM451 cells, compared with a normal human melanocyte cell line, and demonstrated that overexpression of miR-138-5p significantly inhibited the proliferation and invasion of WM451 cells by targeting *HIF-1α* [41]. Additionally, Meng et al. found miR-138-5p significant downregulation in a few blood samples from melanoma patients when compared with healthy control subjects and demonstrated inhibition of cell proliferation and induction of apoptosis with miR-138-5p overexpression in the human melanoma cell line A2058 by targeting *PDK1* [42]. Consistent with these results, we identified higher miR-138-5p and lower *hTERT* expression in human epidermal melanocytes, compared with melanoma cells, observing a negative correlation between them.

In addition, we found that *hTERT* mRNA and protein expressions were increased in pTERTm melanoma cells, compared with nonmutated ones. However, several studies have demonstrated no correlation of hTERT protein expression with mutation status in tumoral tissues, thus suggesting that hTERT protein expression may be regulated by other mechanisms in addition to its promoter mutation [9,12,13,14,15,43]. The discrepancy in *hTERT* expression could be explained by the simplicity of the model used. Cell culture is one of the major tools used in cellular and molecular biology, providing excellent simple model systems for studying the normal physiology and biochemistry of cells. However, it lacks information about interactions between the different cell types as well as between cells and the extracellular matrix involved in the regulation of the in vivo homeostasis.

As previously described in other types of cancer, we identified *hTERT* as a direct target of miR-138-5p in melanoma cells and confirmed a decrease in hTERT protein expression with the enforced overexpression of this miRNA [35]. Thus, the posttranscriptional regulation of the *hTERT* gene by miR-138-5p may have an important role in the telomerase expression of melanoma cells. Thereby, in melanoma samples, this could be a mechanism to approach to abrogate the elevated telomerase activity and telomerase expression in melanoma patients.

Moreover, we observed a significant reduction in telomerase activity, cell proliferation, and migration with miR-138-5p overexpression in A2058 cells. The uncontrolled cell proliferation state that occurs by disruption of the equilibrium between cell death and proliferation is one of the main hallmarks of cancer [44]. Together with migration, they are two key aspects of tumor progression; therefore, its suppression strongly suggested that miR-138-5p downregulation may promote melanoma tumorigenesis.

Identification of molecular alterations implicate in melanoma development and progression may represent potential therapeutic targets [45]. A novel treatment that targets the RNA template of *hTERT* (imetelstad) has been shown to inhibit telomerase activity and cell proliferation in various cancer types [46,47,48]. Although there is still much to understand about the complexity of miRNAs regulation, the discovery of miRNAs that target *hTERT* also could be a promising approach to treat cancers that are telomerase dependent. In this sense, ongoing clinical research is testing miR-based treatments as miR-34a mimic in solid tumors (Phase I, NCT01829971) [49]. Combined treatment with several of these approaches may enhance individual anticancer effects. For instance, melatonin potentiates the vemurafenib-mediated antitumor effect in melanoma through the suppression of the hTERT expression [50].

In conclusion, these preliminary data identified miR-138-5p as a crucial tumor suppressor miRNA involved in telomerase regulation. The administration of miR-138-5p as a combination therapy with immunotherapy or targeted therapies could be used in the treatment of advanced melanoma. However, more preclinical/clinical studies are needed in order to provide a more comprehensive view of miRNA-based therapies.

## Supplementary Materials

The following are available online at https://www.mdpi.com/article/10.3390/genes12121931/s1, Table S1: TERT luciferase reported plasmid, Table S2: GAPDH luciferase reported plasmid, Table S3: Random C1 luciferase reported plasmid.

## References

[1] Bandarchi B., Ma L., Navab R., Seth A., Rasty G. (2010). From melanocyte to metastatic malignant melanoma. Dermatol. Res. Pract..

[2] Little E.G., Eide M.J. (2012). Update on the current state of melanoma incidence. Dermatol. Clin..

[3] Lombardo N., Della Corte M., Pelaia C., Piazzetta G., Lobello N., Del Duca E., Bennardo L., Nistico S.P. (2021). Primary Mucosal Melanoma Presenting with a Unilateral Nasal Obstruction of the Left Inferior Turbinate. Medicina.

[4] Horn S., Figl A., Rachakonda P.S., Fischer C., Sucker A., Gast A., Kadel S., Moll I., Nagore E., Hemminki K. (2013). TERT promoter mutations in familial and sporadic melanoma. Science.

[5] Huang F.W., Hodis E., Xu M.J., Kryukov G.V., Chin L., Garraway L.A. (2013). Highly recurrent TERT promoter mutations in human melanoma. Science.

[6] de Unamuno Bustos B., Murria Estal R., Perez Simo G., Oliver Martinez V., Llavador Ros M., Palanca Suela S., Botella Estrada R. (2016). Lack of TERT promoter mutations in melanomas with extensive regression. J. Am. Acad. Dermatol..

[7] Griewank K.G., Murali R., Puig-Butille J.A., Schilling B., Livingstone E., Potrony M., Carrera C., Schimming T., Moller I., Schwamborn M. (2014). TERT promoter mutation status as an independent prognostic factor in cutaneous melanoma. J. Natl. Cancer Inst..

[8] Heidenreich B., Nagore E., Rachakonda P.S., Garcia-Casado Z., Requena C., Traves V., Becker J., Soufir N., Hemminki K., Kumar R. (2014). Telomerase reverse transcriptase promoter mutations in primary cutaneous melanoma. Nat. Commun..

[9] Populo H., Boaventura P., Vinagre J., Batista R., Mendes A., Caldas R., Pardal J., Azevedo F., Honavar M., Guimaraes I. (2014). TERT promoter mutations in skin cancer: The effects of sun exposure and X-irradiation. J. Investig. Dermatol..

[10] Lee S., Opresko P., Pappo A., Kirkwood J.M., Bahrami A. (2016). Association of TERT promoter mutations with telomerase expression in melanoma. Pigment Cell Melanoma Res..

[11] Vallarelli A.F., Rachakonda P.S., Andre J., Heidenreich B., Riffaud L., Bensussan A., Kumar R., Dumaz N. (2016). TERT promoter mutations in melanoma render TERT expression dependent on MAPK pathway activation. Oncotarget.

[12] de Unamuno Bustos B., Sahuquillo Torralba A., Moles Poveda P., Perez Simo G., Simarro Farinos J., Llavador Ros M., Palanca Suela S., Botella Estrada R. (2019). Telomerase Expression in a Series of Melanocytic Neoplasms. Actas Dermosifiliogr..

[13] Hugdahl E., Kalvenes M.B., Mannelqvist M., Ladstein R.G., Akslen L.A. (2018). Prognostic impact and concordance of TERT promoter mutation and protein expression in matched primary and metastatic cutaneous melanoma. Br. J. Cancer.

[14] Kohli J.S., Mir H., Wasif A., Chong H., Akhras V., Kumar R., Nagore E., Bennett D.C. (2017). ETS1, nucleolar and non-nucleolar TERT expression in nevus to melanoma progression. Oncotarget.

[15] Masui K., Komori T., Kato Y., Masutomi K., Ichimura K., Ogasawara S., Kaneko M.K., Oki H., Suzuki H., Nitta M. (2018). Elevated TERT Expression in TERT-Wildtype Adult Diffuse Gliomas: Histological Evaluation with a Novel TERT-Specific Antibody. Biom. Res. Int..

[16] Diaz A., Puig-Butille J.A., Munoz C., Costa D., Diez A., Garcia-Herrera A., Carrera C., Badenas C., Sole F., Malvehy J. (2014). TERT gene amplification is associated with poor outcome in acral lentiginous melanoma. J. Am. Acad. Dermatol..

[17] Zhang C., Song C., Liu T., Tang R., Chen M., Gao F., Xiao B., Qin G., Shi F., Li W. (2017). KMT2A promotes melanoma cell growth by targeting hTERT signaling pathway. Cell Death Dis..

[18] Fan Y., Lee S., Wu G., Easton J., Yergeau D., Dummer R., Vogel P., Kirkwood J.M., Barnhill R.L., Pappo A. (2016). Telomerase Expression by Aberrant Methylation of the TERT Promoter in Melanoma Arising in Giant Congenital Nevi. J. Investig. Dermatol..

[19] Guilleret I., Yan P., Grange F., Braunschweig R., Bosman F.T., Benhattar J. (2002). Hypermethylation of the human telomerase catalytic subunit (hTERT) gene correlates with telomerase activity. Int. J. Cancer.

[20] Qin Y.Z., Xie X.C., Liu H.Z., Lai H., Qiu H., Ge L.Y. (2015). Screening and preliminary validation of miRNAs with the regulation of hTERT in colorectal cancer. Oncol. Rep..

[21] Hrdlickova R., Nehyba J., Bargmann W., Bose H.R. (2014). Multiple tumor suppressor microRNAs regulate telomerase and TCF7, an important transcriptional regulator of the Wnt pathway. PLoS ONE.

[22] Ambros V. (2004). The functions of animal microRNAs. Nature.

[23] de Unamuno B., Palanca S., Botella R. (2015). Update on melanoma epigenetics. Curr. Opin. Oncol..

[24] Bartel D.P. (2004). MicroRNAs: Genomics, biogenesis, mechanism, and function. Cell.

[25] Bartels C.L., Tsongalis G.J. (2009). MicroRNAs: Novel biomarkers for human cancer. Clin. Chem..

[26] Nair V.S., Maeda L.S., Ioannidis J.P. (2012). Clinical outcome prediction by microRNAs in human cancer: A systematic review. J. Natl. Cancer Inst..

[27] Wang Y., Lee C.G. (2009). MicroRNA and cancer—Focus on apoptosis. J. Cell Mol. Med..

[28] Chen J., Feilotter H.E., Pare G.C., Zhang X., Pemberton J.G., Garady C., Lai D., Yang X., Tron V.A. (2010). MicroRNA-193b represses cell proliferation and regulates cyclin D1 in melanoma. Am. J. Pathol..

[29] Xu Y., Brenn T., Brown E.R., Doherty V., Melton D.W. (2012). Differential expression of microRNAs during melanoma progression: miR-200c, miR-205 and miR-211 are downregulated in melanoma and act as tumour suppressors. Br. J. Cancer.

[30] Chai L., Kang X.J., Sun Z.Z., Zeng M.F., Yu S.R., Ding Y., Liang J.Q., Li T.T., Zhao J. (2018). MiR-497-5p, miR-195-5p and miR-455-3p function as tumor suppressors by targeting hTERT in melanoma A375 cells. Cancer Manag. Res..

[31] Ohira T., Naohiro S., Nakayama Y., Osaki M., Okada F., Oshimura M., Kugoh H. (2015). miR-19b regulates hTERT mRNA expression through targeting PITX1 mRNA in melanoma cells. Sci. Rep..

[32] Song G., Wang R., Guo J., Liu X., Wang F., Qi Y., Wan H., Liu M., Li X., Tang H. (2015). miR-346 and miR-138 competitively regulate hTERT in GRSF1- and AGO2-dependent manners, respectively. Sci. Rep..

[33] Wang X., Zhao Y., Cao W., Wang C., Sun B., Chen J., Li S., Chen J., Cui M., Zhang B. (2017). miR-138-5p acts as a tumor suppressor by targeting hTERT in human colorectal cancer. Int. J. Clin. Exp. Pathol..

[34] Zhang X.L., Xu L.L., Wang F. (2017). Hsa_circ_0020397 regulates colorectal cancer cell viability, apoptosis and invasion by promoting the expression of the miR-138 targets TERT and PD-L1. Cell Biol. Int..

[35] Mitomo S., Maesawa C., Ogasawara S., Iwaya T., Shibazaki M., Yashima-Abo A., Kotani K., Oikawa H., Sakurai E., Izutsu N. (2008). Downregulation of miR-138 is associated with overexpression of human telomerase reverse transcriptase protein in human anaplastic thyroid carcinoma cell lines. Cancer Sci..

[36] Livak K.J., Schmittgen T.D. (2001). Analysis of relative gene expression data using real-time quantitative PCR and the 2(-Delta Delta C(T)) Method. Methods.

[37] Cho W.C. (2007). OncomiRs: The discovery and progress of microRNAs in cancers. Mol. Cancer.

[38] Liu X., Wang C., Chen Z., Jin Y., Wang Y., Kolokythas A., Dai Y., Zhou X. (2011). MicroRNA-138 suppresses epithelial-mesenchymal transition in squamous cell carcinoma cell lines. Biochem. J..

[39] Liu X., Jiang L., Wang A., Yu J., Shi F., Zhou X. (2009). MicroRNA-138 suppresses invasion and promotes apoptosis in head and neck squamous cell carcinoma cell lines. Cancer Lett..

[40] Chen L., Lu M.H., Zhang D., Hao N.B., Fan Y.H., Wu Y.Y., Wang S.M., Xie R., Fang D.C., Zhang H. (2014). miR-1207-5p and miR-1266 suppress gastric cancer growth and invasion by targeting telomerase reverse transcriptase. Cell Death Dis..

[41] Chen Y., Cao K.E., Wang S., Chen J., He B., He G.U., Chen Y., Peng B., Zhou J. (2016). MicroRNA-138 suppresses proliferation, invasion and glycolysis in malignant melanoma cells by targeting HIF-1alpha. Exp. Ther. Med..

[42] Meng F., Zhang Y., Li X., Wang J., Wang Z. (2017). Clinical significance of miR-138 in patients with malignant melanoma through targeting of PDK1 in the PI3K/AKT autophagy signaling pathway. Oncol. Rep..

[43] Salgado C., Roelse C., Nell R., Gruis N., van Doorn R., van der Velden P. (2020). Interplay between TERT promoter mutations and methylation culminates in chromatin accessibility and TERT expression. PLoS ONE.

[44] Sandal T. (2002). Molecular aspects of the mammalian cell cycle and cancer. Oncologist.

[45] Rapanotti M.C., Cugini E., Nuccetelli M., Terrinoni A., Di Raimondo C., Lombardo P., Costanza G., Cosio T., Rossi P., Orlandi A. (2021). MCAM/MUC18/CD146 as a Multifaceted Warning Marker of Melanoma Progression in Liquid Biopsy. Int. J. Mol. Sci..

[46] Salloum R., Hummel T.R., Kumar S.S., Dorris K., Li S., Lin T., Daryani V.M., Stewart C.F., Miles L., Poussaint T.Y. (2016). A molecular biology and phase II study of imetelstat (GRN163L) in children with recurrent or refractory central nervous system malignancies: A pediatric brain tumor consortium study. J. Neurooncol..

[47] Tefferi A., Lasho T.L., Begna K.H., Patnaik M.M., Zblewski D.L., Finke C.M., Laborde R.R., Wassie E., Schimek L., Hanson C.A. (2015). A Pilot Study of the Telomerase Inhibitor Imetelstat for Myelofibrosis. N. Engl. J. Med..

[48] Wu X., Zhang J., Yang S., Kuang Z., Tan G., Yang G., Wei Q., Guo Z. (2017). Telomerase antagonist imetelstat increases radiation sensitivity in esophageal squamous cell carcinoma. Oncotarget.

[49] Rupaimoole R., Slack F.J. (2017). MicroRNA therapeutics: Towards a new era for the management of cancer and other diseases. Nat. Rev. Drug Discov..

[50] Hao J., Fan W., Li Y., Tang R., Tian C., Yang Q., Zhu T., Diao C., Hu S., Chen M. (2019). Melatonin synergizes BRAF-targeting agent vemurafenib in melanoma treatment by inhibiting iNOS/hTERT signaling and cancer-stem cell traits. J. Exp. Clin. Cancer Res. CR.

